# Process Mapping in a Pediatric Emergency Department to Minimize Missed Urinary Tract Infections

**DOI:** 10.1155/2016/2625870

**Published:** 2016-11-16

**Authors:** Morgan Black, Valene Singh, Vladimir Belostotsky, Madan Roy, Deborah Yamamura, Kathryn Gambarotto, Keith Lau, April J. Kam

**Affiliations:** ^1^McMaster Family Practice, Department of Family Medicine, McMaster University, 3rd Floor, 100 Main St. W., Hamilton, ON, Canada L8P 1H6; ^2^University of Toronto Faculty of Medicine, 1 King's College Cir No. 3172, Toronto, ON, Canada M5S 1A8; ^3^Division of Pediatric Nephrology, McMaster Children's Hospital, 1280 Main Street West, Room 3A, Hamilton, ON, Canada L8S 4K1; ^4^Division of General Pediatrics, McMaster Children's Hospital, 1280 Main Street West, Room 3A, Hamilton, ON, Canada L8S 4K1; ^5^Division of Medical Microbiology, Hamilton General Hospital, Room W-1, I-90, 237 Barton St. East, Hamilton, ON, Canada L8L 2X2; ^6^The University of Hong Kong-Shenzhen Hospital, 1 Haiyuan 1st Road, Futian District, Shenzhen, Guangdong 518053, China; ^7^Division of Pediatric Emergency Medicine, McMaster Children's Hospital, 1280 Main Street West, Room 2N49, Hamilton, ON, Canada L8S 4K1

## Abstract

Urinary tract infections (UTIs) are common in young children and are seen in emergency departments (EDs) frequently. Left untreated, UTIs can lead to more severe conditions. Our goal was to undertake a quality improvement (QI) initiative to help minimize the number of children with missed UTIs in a newly established tertiary care pediatric emergency department (PED). A retrospective chart review was undertaken to identify missed UTIs in children < 3 years old who presented to a children's hospital's ED with positive urine cultures. It was found that there was no treatment or follow-up in 12% of positive urine cultures, indicating a missed or possible missed UTI in a significant number of children. Key stakeholders were then gathered and process mapping (PM) was completed, where gaps and barriers were identified and interventions were subsequently implemented. A follow-up chart review was completed to assess the impact of PM in reducing the number of missed UTIs. Following PM and its implementation within the ED, there was no treatment or follow-up in only 1% of cases. Based on our results, the number of potentially missed UTIs in the ED decreased dramatically, indicating that PM can be a successful QI tool in an acute care pediatric setting.

## 1. Introduction

Approximately 8% of girls and 2% of boys will have had a urinary tract infection (UTI) by the age of 8 [1]. It is important to appropriately diagnose and actively manage UTIs because of the potential long-term morbidity [2–5]. If the diagnosis of a UTI is missed, renal damage may be exacerbated [2]. As the risk for renal scarring is highest in children under 4 years of age, it is important to identify and treat UTIs in infants and young children in a timely manner [6].

High volume, complex services such as pediatric emergency departments (PED) are at risk of inefficiency. To date, there is a lack of research that specifically addresses the frequency of missed UTI diagnoses in children and no previously published studies which specifically looked at the rate of missed urinary tract infections in the PED. The importance of reducing laboratory postanalytical errors such as delayed or missed reaction to laboratory reporting by improving processes is increasingly recognized [7].

Lean is a system that encompasses key principles to evaluate a process or procedure and identify valuable and nonvaluable components of a process. Process mapping (PM) is an analytical tool applied for process improvement and is often a first step in implementing Lean methodology. Stakeholders in the process are initially gathered to establish the current state of a process. A process map will then be generated and used to identify areas for immediate analysis and intervention. Areas within the process that both add value and remove value are identified. Those that do not add value are removed, and portions that do add value are kept and at times corrected if inadequate. This analysis defines metrics for the stakeholders [8]. After intervention, new performance is assessed by direct data evaluation. The process is completed with an analysis of effect and plans made for addressing future focus areas. Lean process improvement through PM can reduce health care waste and improve patient safety [8].

## 2. Materials and Methods

McMaster Children's Hospital is an urban, university-affiliated, tertiary care pediatric hospital in Hamilton, Ontario, Canada. The PED sees approximately 45,000 children annually. At the opening of the ED, there was not yet an established protocol or system in place to systematically manage and follow up positive urine cultures identified within the ED. This quality improvement (QI) study was comprised of an initial retrospective chart review within the McMaster Children's Hospital ED to collect baseline data and to review current practices within the ED with regard to managing UTIs in young children. This was followed by a QI strategy, which included PM through Lean methodology. Our interventions were subsequently evaluated by undertaking a second identical retrospective chart review. Each part of this stepwise process is outlined in further detail below.

During the initial chart review from January to December 2011, charts were reviewed for all children < 3 years old presenting to the McMaster Children's ED with positive urine cultures (defined as ≥10^4^ CFU/mL). Exclusion criteria for the chart review included patients seen in the ED by a pediatric subspecialist (as they were not evaluated by a PED physician and disposition was determined by the subspecialist), specimen collected by bag sample (due to high risk of contamination and false positives), and patients admitted to hospital at original visit.

Two research student volunteers reviewed electronic charts and completed the data collection forms on an Excel database. The ED uses paper charts which are then scanned into electronic medical records. Laboratory results are available electronically but the ED is notified through paper copies. Although a number of parameters were recorded on the data collection form, the main outcomes of interest were whether appropriate antibiotics were given and/or appropriate follow-up was arranged. Follow-up was determined by reviewing the patients' scanned medical charts and noting whether there was a follow-up appointment with the Pediatric Emergency Referral Clinic (PERC), return to the ED, or a phone call to the patient's family regarding the positive urine culture.

Following the initial chart review, the data was presented to members of the ED staff and study group members. A discussion was facilitated to determine future directions in terms of improving process so that the proportion of missed UTI diagnoses and follow-ups could be decreased. This is where the Lean methodology for PM was implemented. After gathering together key stakeholders operating within the ED, a working group (WG) was created to identify areas for improvement in UTI diagnosis and management. WG team members included ED management, ED physicians, ED nurses, audit group representative, microbiology representative, and a quality control officer. The WG all participated in PM led by a team member trained in Lean methodology.

**Figure 1:**
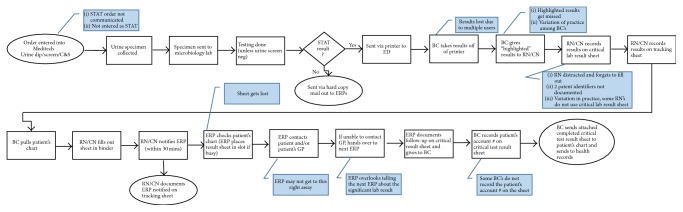
Process map of significant urine culture result notification process derived by working group and used in the McMaster Children's Hospital Emergency Department. BC: business clerk; ERP: Emergency Room Physician.

Numerous interventions were proposed by the WG based on the gaps identified through the method of PM. The interventions and key players are outlined in Table 1. The revised process maps based on these interventions were distributed and email reminders outlining the exact protocol for the positive urine culture notification process were sent out to all involved individuals (shown in Figure 1). Figure 1 outlines the revised notification process but also makes note of the gaps identified during the PM process. These interventions were all practically implemented into the McMaster Children's Hospital ED by June 2013.

As part of the Plan Do Study Act (PDSA) cycle and in order to adequately assess how the proposed interventions impacted the number of missed urinary tract infections, an identical chart review was completed from July to December 2013 after all of the proposed interventions had been implemented. The same inclusion and exclusion criteria and the same data collection sheet were utilized in the follow-up study to create identical parameters as the preintervention chart review. In this follow-up chart review, 114 charts were reviewed and, after applying exclusion criteria, 80 charts were available for final review. Once again, the main outcome of interest was whether appropriate antibiotics were given and/or appropriate follow-up was arranged.

## 3. Statistics

In order to ensure adequate interrater reliability when performing the initial retrospective chart review with the research volunteers, a kappa value was calculated using Stata 10.1. The kappa statistic is a unique percent agreement when calculating interrater agreement as it takes into account the agreement that can occur just based on chance alone. A kappa score of >0.8 indicates almost perfect agreement.

## 4. Ethical Considerations

Both the initial study and follow-up study were retrospective chart reviews. Patient charts with positive urine cultures were reviewed and data included general visit information, urine parameters, documented antibiotic treatment, and follow-up. Hamilton integrated Research Ethics Board (HiREB) approval was obtained.

## 5. Results

In the initial chart review, 159 charts from patients < 3 years old from January to December 2011 were reviewed. Based on the established inclusion/exclusion criteria, 82 were eligible for final review. The kappa values for admission, antibiotic implementation, and follow-up were 0.87, 0.83, and 0.84, respectively, indicating almost perfect agreement. A breakdown of the final results in terms of follow-up and treatment is shown in Table 2(a).

The results of the chart review indicated that for 38 cases (46%), antibiotics were given and a follow-up was arranged with the pediatrician, Pediatric Emergency Referral Clinic (PERC), or the family doctor. In 14 (17%) cases, antibiotics were given but a follow-up was not arranged. In 15 (18%) cases, antibiotics were not given but a follow-up was arranged with either the pediatrician, PERC, or the family doctor. The number this study is most concerned about is the 15 cases (18%) where antibiotics were not given and a follow-up was not arranged. After further detailed review by the investigating physicians of these 15 cases, 5 either did not have a UTI or had a follow-up, 4 cases had a possible UTI, and 6 did have a UTI. This indicated a total miss or possible miss of 10 cases (12%). Furthermore, UTI was indicated as the discharge diagnosis in 45/52 cases in the treated group (87%) and in 3/30 cases in the untreated group (10%).

As the interventions derived from PM were fully implemented within the McMaster Children's Emergency Department by June 2013, the follow-up chart review began in July 2013. In the follow-up chart review that was completed from July to December 2013, 114 charts were reviewed. Based on the same specified inclusion/exclusion criteria, 80 charts were eligible for final review. Of these 80 cases, it was found that were was no treatment or follow-up in only 1% of cases (Table 2(b)).

## 6. Discussion

PM is a method of depicting a process or information flow in a diagrammatic form. It provides a step-by-step representation of a particular process to achieve a certain outcome and identifies both key players and activities within a certain process or service. In order to effectively process map, it was important that a WG was created so that members involved in each area could contribute insight into their particular role, cognitive processes, and actions and help gain insight into how colleagues perceive the same tasks. One key to PM for QI is that it is important to map the current process and not the desired process, such that areas for improvement can be identified and solutions can be implemented to fill these gaps. By doing this, one can gain a clear understanding of the existing state and changes can be made to improve a service. By utilizing this method, our WG was able to successfully create a revised process map and implement proposed interventions practically within our ED.

Although PM has demonstrated performance improvement in other healthcare processes, such as the trauma discharge process, no literature has looked at its value within an emergency department setting [8]. Following implementation of the interventions devised by the interprofessional team through PM, the number of missed UTIs in children < 3 years old was decreased from 12% to 1%. It should be noted that the one case of concern in the follow-up study was actually a patient who subsequently returned to the ED before their urine culture results were back for review. This patient was subsequently admitted and had appropriate inpatient care. Therefore, despite this being the most appropriate category to include this patient, it might not necessarily have been a true missed urinary tract infection had the ED received the culture results and sensitivities and acted on them prior the return of the patient to the ED.

The initial chart review was undertaken over a 12-month period whereas the follow-up chart review following PM was conducted over a 6-month period. Despite the follow-up chart review being only half the time, there were 114 positive urine cultures to review and 80 eligible cases compared to 159 positive urine cultures and 82 eligible cases to review in the initial study. This indicates that during the follow-up chart review period of only 6 months, the number of patients with positive urine cultures approached that of the initial 12-month chart review, indicating a high volume of culture positive urine tests in the 6-month period following PM and implemented interventions. There were no other UTI educational awareness programs implemented in the PED at that time. Despite this high volume, the number of positive cultures missed to both treatment and follow-up remained limited to a single case, reinforcing the effectiveness of PM to reduce UTIs in the PED.

A potential limitation of this study is that the initial chart review was completed when the ED was recently redeveloped and in its early stages of establishment within the community. While the missed UTI rate could have decreased as a result of the ED maturing and improving all its processes overall, we think this is unlikely, since other more established EDs in the area have requested for consultation on the PM process in order to improve their own rates of missed UTIs.

## 7. Conclusions

Overall, as demonstrated with the management of UTI in a newly developed PED, PM as a QI initiative is a practical way to implement quality assurance changes. PM in pediatric emergency medicine is a viable tool for quality improvement. When applied to a newly developed PED, PM identifies areas ripe for improvement. Strategies arising from the input of all stakeholders led to real and identified areas for ongoing improvement. This optimizes resource use and identifies areas for improvement in patient care.

## Figures and Tables

**Table 1:** Interventions Implemented in ED Following Process Mapping.

Players Involved	Intervention
Lab	Microbiology Lab actively sent all ED based results to the ED
ED	All microbiology results come to specific printer in ED
ED-RN	All results sorted by Registered Nurse (RN)
ED-Business Clerk	All positive results given to Business Clerk to attach to old chart
ED-Business Clerk	All positive results placed in PED MD folder
ED-Physician	All ED physicians on Day shift check the Positive Results folder and call patient/parents/primary care physician as necessary
ED	All pending sensitivities for positive urine cultures placed in Pending Sensitivities folder
ED	Once sensitivities results matched – charts moved to Completed folder to be taken back by Info Clerk and sent to Sovera (software system where all hospital documentation is scanned, charted and sorted) to be copied

**(a):** 

	Antibiotics given in ER	No antibiotics given in ER	Total
Follow-up	38	15	53
No follow-up	14	**15**	29

	52	30	82

**(b):** 

	Antibiotics given in ER	No antibiotics given in ER	Total
Follow-up	60	16	76
No follow-up	3	**1**	4

	63	17	80
